# Development and Validation of Global Leadership Initiative on Malnutrition for Prognostic Prediction in Patients Who Underwent Cardiac Surgery

**DOI:** 10.3390/nu14122409

**Published:** 2022-06-09

**Authors:** Zhang Liu, Zile Shen, Wangfu Zang, Jian Zhou, Zhen Yu, Peng Zhang, Xialin Yan

**Affiliations:** 1 Department of Cardio-Thoracic Surgery, Shanghai Tenth People’s Hospital, School of Medicine, Tongji University, Shanghai 200072, China; 1910858@tongji.edu.cn (Z.L.); zangwf@tongji.edu.cn (W.Z.); drzhoujian1368@163.com (J.Z.); 2 Department of Gastrointestinal Surgery, Shanghai Tenth People’s Hospital, School of Medicine, Tongji University, Shanghai 200072, China; 1910857@tongji.edu.cn (Z.S.); yuzhen@tongji.edu.cn (Z.Y.); 3 Department of Colorectal Anal Surgery, The First Affiliated Hospital of Wenzhou Medical University, Wenzhou 325000, China

**Keywords:** Global Leadership Initiative on Malnutrition, cardiac surgery, nomogram, clinical outcomes

## Abstract

The Global Leadership Initiative on Malnutrition (GLIM) has achieved a consensus for the diagnosis of malnutrition in recent years. This study aims to determine the prognostic effect of the GLIM after cardiac surgery. A total of 603 patients in the training cohort and 258 patients in the validation cohort were enrolled in this study. Perioperative characteristics and follow-up data were collected. A nomogram based on independent prognostic predictors was developed for survival prediction. In total, 114 (18.9%) and 48 (18.6%) patients were defined as being malnourished according to the GLIM criteria in the two cohorts, respectively. Multivariate regression analysis showed that GLIM-defined malnutrition was an independent risk factor of total complication (OR 1.661, 95% CI: 1.063–2.594) and overall survival (HR 2.339, 95% CI: 1.504–3.637). The c-index was 0.72 (95% CI: 0.66–0.79) and AUC were 0.800, 0.798, and 0.780 for 1-, 2-, and 3-year survival prediction, respectively. The calibration curves of the nomogram fit well. In conclusion, GLIM criteria can efficiently identify malnutrition and has a prognostic effect on clinical outcomes after cardiac surgery. GLIM-based nomogram has favorable performance in survival prediction.

## 1. Introduction

As a global public health problem, malnutrition is a major concern in cardiothoracic surgery. Growing evidence suggests that malnutrition significantly affects postoperative recovery in patients who underwent cardiac surgery, and these patients with malnutrition tend to have longer postoperative hospital stays, longer intensive care unit (ICU) stays, and poorer long-term outcomes [1,2,3]. Although there is an increasing awareness of the vital function of malnutrition in determining postoperative outcomes in patients who underwent cardiac surgery, the current predominant tool predicting postoperative survival before cardiac surgery [4], the European System for Cardiac Operative Risk Evaluation II (EuroSCORE II) [5], does not take nutritional index into consideration.

Recently, the Global Leadership Initiative on Malnutrition (GLIM) has reached a global consensus on malnutrition diagnosis in clinical settings [6]. The GLIM is a two-step approach to diagnose malnutrition, which consists of phenotypic and etiological diagnoses. Recent studies have demonstrated the effectiveness of the GLIM in identifying malnutrition and predicting prognosis among various clinical contexts [7,8,9].

Several predictive models involving the GLIM have been developed to optimize nutritional-status-related prognostic assessment of different patients [10,11,12]. However, for cardiothoracic patients, there were few such studies. Therefore, our study is the first study to validate the GLIM in patients who underwent cardiac surgery. We also investigated the role of the GLIM in predicting short- and long-term outcomes after cardiac surgery. In addition, a nomogram model was developed to refine the GLIM for predicting long-term survival in cardiac surgery patients.

## 2. Materials and Methods

### 2.1. Patients

This retrospective, observational study was conducted at the Department of Cardio-Thoracic Surgery, Shanghai Tenth People’s Hospital (registration number: ChiCTR2200056468) from December 2015 to March 2021. All patients who underwent coronary artery bypass grafting (CABG) and/or valve surgery through midline sternotomy were eligible for this study. The inclusion criteria were as follows: (1) Age ≥18 years old; (2) underwent first major cardiac surgery via midline sternotomy; (3) available for nutritional screening on admission and preoperative chest computed tomography (CT) images. Emergency surgery patients were excluded from this study. This study was conducted according to the guidelines of the Declaration of Helsinki, and ethical approval was obtained from the Ethics Committee of Shanghai Tenth People’s Hospital.

### 2.2. Data Collection

The following data were prospectively collected including (1) preoperative baseline, including general information, cardiac function-related information, laboratory data, existing comorbidity, and medical history, which were collected within 48 h after admission; (2) operative details, including surgical type, type of involved valves, number of bypassed vessels, operative time, cardiopulmonary bypass (CPB) time, and aortic cross-clamp time; (3) short-term postoperative complications within 30 days of operation, which were classified according to the Clavien-Dindo classification. Only complications classified as grade II or above were analyzed.

### 2.3. Follow Up

Long-term outcomes were acquired by telephone interviews or outpatient visits, which were performed 1 month after surgery, and then every 3 months for the first 2 years, and every 6 months after that. The last follow-up date was 31 January 2022. Overall survival (OS) was calculated from the date of surgery to the date of death from any cause.

### 2.4. Muscle Mass Measurements

Preoperative chest CT images at the 12th thoracic vertebra (T12) level were processed by INFINITT PACS software (version 3.0.11.3, Seoul, Korea) for obtaining muscle mass. Skeletal muscle tissues were identified by a Hounsfield unit (HU) thresholds range of −29 to +150 HU and normalized by height (m^2^) to acquire T12 SMI (cm^2^/m^2^). The total skeletal muscles at T12 level contained the rectus abdominis, external oblique, internal oblique, latissimus dorsi, intercostal, and erector spinae muscles. Consistent with our previous study, cutoff values of T12 SMI were referenced from a large-scale study, which were 28.8 cm^2^/m^2^ for male and 20.8 cm^2^/m^2^ for female, respectively [13,14].

### 2.5. Assessment of Nutritional Status

According to the GLIM criteria, we defined malnutrition using a two-step approach. First, all the patients received nutritional screening by Malnutrition Universal Screening Tool (MUST), and a patient with a MUST score ≥1 was considered at risk of malnutrition. Then, the combined criteria were required to confirm the diagnosis of malnutrition, which consisted of at least one of the three phenotypic criteria (non-volitional weight loss, low body mass index [BMI], and reduced muscle mass) and at least one of the two etiologic criteria (reduced food intake or assimilation, and inflammation or disease burden). Since patients who underwent CABG and/or valve surgery had already met one etiologic criterion (disease burden) [15,16], we diagnosed malnutrition based on the phenotypic criteria in the present study.

For the phenotypic criteria, non-volitional weight loss was defined in patients with unintentional weight loss >5% within the past 6 months, or >10% beyond 6 months; BMI < 18.5 kg/m^2^ was defined as low BMI if patients were younger than 70 y, and BMI < 20 kg/m^2^ was defined as low BMI for those aged 70 y or older [6]. Muscle mass was evaluated by SMI, and calculated from chest CT images, which has been described above.

### 2.6. Statistical Analysis

Continuous variables with a normal distribution were expressed as means and standard deviations (SDs) and compared using the Student’s *t*-test. Quantitative variables with non-normal distribution were expressed as medians and interquartile ranges (IQRs) and compared using the Mann–Whitney U test. Categorical variables were expressed as numbers and proportions and compared using Chi-squared or Fisher’s exact test. The training cohort and validation cohort were obtained by random resampling with a 70/30 split ratio. Risk factors for postoperative complication in the training cohort were assessed by univariate analysis. Factors with *p* < 0.1 were included in the multivariate analysis and forward stepwise selection methodology was performed. Kaplan–Meier curves and the Cox proportional hazards model were performed to analyze long-term survival. Based on the result of the multivariate Cox regression, a nomogram was formulated to predict 1-, 2-, and 3-year OS rates. The discriminative ability and predictive accuracy were evaluated by c-index, the area under receiver operating characteristic curve (AUC) and calibration curve. Statistical significance was set at a 2-tailed *p* value < 0.05. All statistical analysis was performed with SPSS software version 26 (Armonk, NY, USA) and R version 4.1.3 (Vienna, Austria).

## 3. Results

### 3.1. Baseline Characteristics

Between December 2015 and March 2021, a total of 917 patients underwent CABG and/or valve surgery in our institution. In total, 56 patients who did not meet the inclusion criteria were excluded, and all remaining 861 patients agreed to be enrolled in this study (Figure S1 in Supplementary Materials). Patients were randomly split into the training cohort (*n* = 603) or the validation cohort (*n* = 258). Overall, 114 (18.9%) and 48 (18.6%) patients were defined as being malnourished according to the GLIM criteria in the training cohort and the validation cohort, respectively (Table 1). Reduced muscle mass was the most common phenotypic criterion of the GLIM among patients who underwent cardiac surgery.

The preoperative characteristics of the study subjects are shown in Table 2. The preoperative baseline of patients in the malnutrition group and non-malnutrition group were comparable. Patients with malnutrition had lower BMI, red blood cells, hemoglobin, lymphocytes, and higher C-reactive protein and platelet-to-lymphocyte ratio than patients without malnutrition in both cohorts. Table 3 presents the intraoperative characteristics of study subjects. The mean operative time (211 [IQR 57] vs. 215 [IQR 61] min, 214 [IQR 60] vs. 213 [IQR 56] min) and CPB time (80.5 [IQR 52] vs. 81 [IQR 54] min, 76 [IQR 52] vs. 82 [IQR 41] min) were comparable between patients with and without malnutrition in both cohorts.

### 3.2. Relationship between GLIM-Defined Malnutrition and Short-Term Outcomes

Postoperative complications occurred in 268 (44.4%) of the patients in the training cohort (Table 4). Patients with malnutrition tended to have higher incidence of postoperative complications (53.5% vs. 42.3%, *p* = 0.031) and prolonged ICU stays (21.9% vs. 13.7%, *p* = 0.028) than patients without malnutrition. This may lead to higher medical costs (131,751 [IQR 56,718] vs. 130,661 [IQR 42,482] CNY, *p* = 0.084), though without statistical significance. GLIM-defined malnutrition showed a significant relationship with postoperative complication in the univariate and multivariate logistic regression (Table 5). Additionally, age (*p* < 0.001), gender (female, *p* = 0.006), left ventricular ejection (LVEF) ≤ 50% (*p* = 0.001), EuroSCORE II ≥ 4% (*p* = 0.013), cerebrovascular disease (*p* = 0.039), and operative time (*p* < 0.001) remained to be independent risk factors of postoperative complication.

### 3.3. Relationship between GLIM-Defined Malnutrition and OS

**Figure 1:**
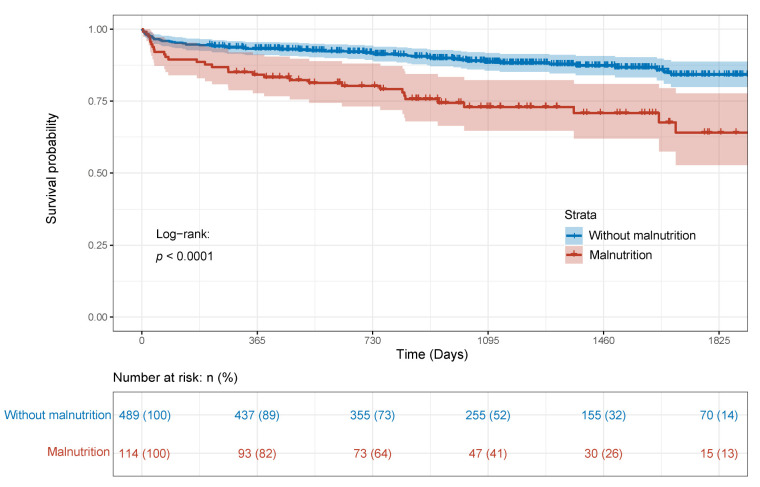
Kaplan–Meier curve of overall survival stratified by GLIM criteria.

Over a median of 3.34 years (IQR 2.20–4.52) follow-up period, 89 (14.8%) patients died in the training cohort. Figure 1 demonstrated that patients with malnutrition had a worse OS than patients without malnutrition in Kaplan–Meier curves (Log-rank: *p* < 0.0001). Clinical variables and serum markers were compared in the Cox regression (Table 6). After multivariable adjustment by various parameters including age (*p* < 0.001), cerebrovascular disease (*p* = 0.009), and CPB time (*p* < 0.001), GLIM-defined malnutrition remained to be an independent prognostic factor for OS in patients who underwent cardiac surgery in the training cohort (*p* < 0.001).

### 3.4. Development and Validation of a Prognostic Nomogram

**Figure 2:**
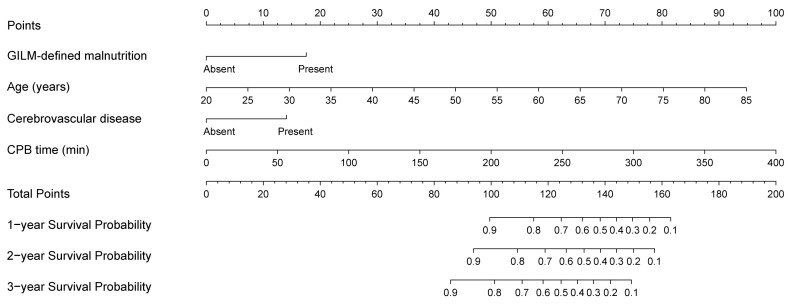
A nomogram model for survival prediction. CPB: cardiopulmonary bypass.

**Figure 3:**
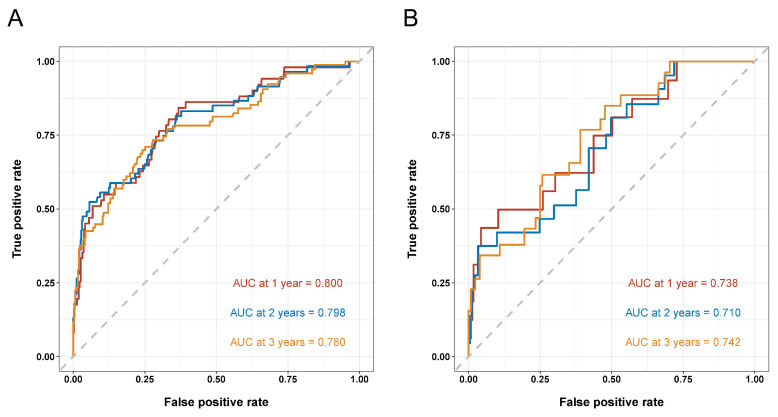
Area under the ROC curves (AUC) for survival prediction in the training cohort (**A**) and validation cohort (**B**). ROC: receiver operator characteristic.

**Figure 4:**
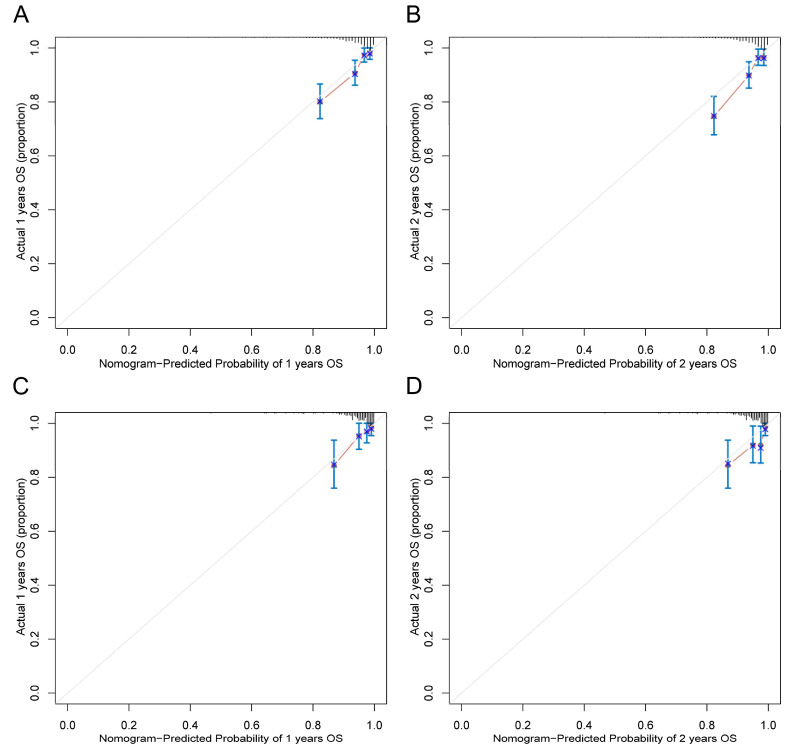
The calibration curve for survival prediction at 1 (**A**), 2 years (**B**) in the training cohort and at 1 (**C**), 2 (**D**) years in the validation cohort.

**Figure 5:**
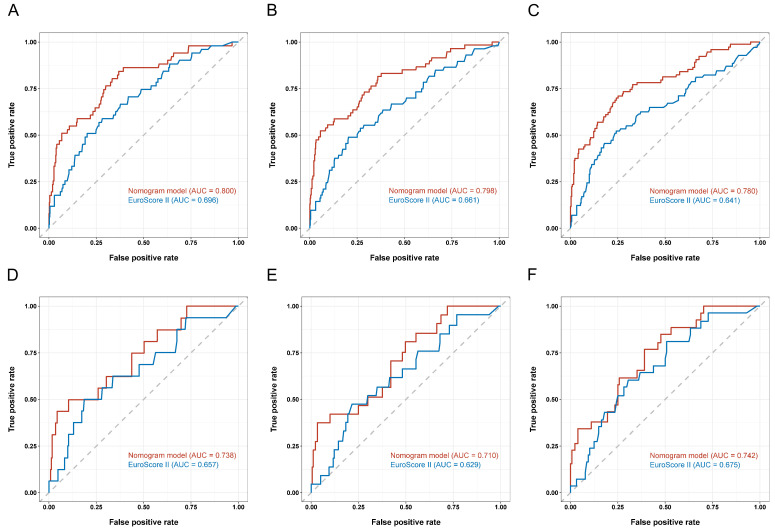
Area under the ROC curves (AUC) of EuroSCORE II and the nomogram for survival prediction at 1 (**A**), 2 (**B**), or 3 (**C**) years in the training cohort and at 1 (**D**), 2 (**E**), and 3 (**F**) years in the validation cohort. ROC: receiver operator characteristic.

A nomogram integrating four selected independent predictors in Table 6 was developed for OS prediction (Figure 2). The points scale of each variable in the nomogram was summed to a total score, which was projected on the bottom scale to indicate the probability of 1-, 2-, and 3-year survival. C-index of the nomogram was 0.72 (95% CI: 0.66–0.79) in the training cohort and 0.72 (95%CI: 0.63–0.82) in the validation cohort. Furthermore, the nomogram yielded an AUC of 0.800, 0.798, and 0.780 in the training cohort and 0.738, 0.710, and 0.742 in the validation cohort for predicting 1-, 2-, and 3-year OS (Figure 3A,B). The calibration curves of the nomogram revealed good agreement between predicted OS and actual observed survival in 1 and 2 years (Figure 4A–D). We also compared the nomogram with EuroSCORE II, and the predictive ability in long-term survival of the nomogram was higher (Figure 5A–F). Taken together, the results demonstrated that the nomogram had obvious distinguishing and calibration performance.

## 4. Discussion

To our knowledge, this is the first study to develop and validate the prognostic effect of the GLIM in patients who underwent cardiac surgery. This present study identified that the incidence of GLIM-defined malnutrition was 18.8% in our population. GLIM-defined malnutrition resulted in higher incidence of postoperative complications and prolonged ICU stays. Compared to patients without malnutrition, patients with malnutrition had poorer OS after cardiac surgery. A nomogram containing the GLIM as well as variables such as CPB time and age was developed. C-index, receiver operator characteristic (ROC) curves and calibration curves showed that our nomogram has good performance in survival prediction.

The stress response caused by surgical trauma leads to hyperglycemia and whole-body protein catabolism [17,18]. Recovery during the postoperative period is always accompanied by increasing protein demands, meeting the needs of wound healing, functional recovery, and proteins synthesis contributed to the immune response [19]. Assessing preoperative nutritional status and identifying patients with malnutrition have important implications for guiding nutritional support over the perioperative period [20]. Patients who undergo cardiac surgery usually suffer greater surgical trauma than patients who undergo other surgical procedures. Especially for the patients who require CPB, life-threatening complications triggered by systemic inflammatory response syndrome are more likely to occur among them, and additional nutritional support is necessary for those patients [21]. The ESPEN guidelines suggest that clinical assessment should be performed to identify malnutrition, which is vital to guide nutritional intervention and improve postoperative outcomes [18,22]. Since cardiac surgical candidates who are malnourished have shown worse clinical outcomes [4,23], early detection of malnutrition and nutritional intervention can significantly benefit them. Therefore, our study focused on using the GLIM to screen patients who need nutritional intervention and verifying the prognostic impact of GLIM-defined malnutrition in patients who underwent cardiac surgery. A prognostic model was also established for risk stratification.

Studies have shown that malnutrition has negative effects on physical function and that it has a close relationship with adverse events, longer hospitalization, and mortality [24,25,26]. Lee et al. showed that malnutrition leads to significantly poorer 1-year survival among patients who underwent transcatheter aortic valve replacement, with an HR of 3.77 (95% CI: 1.54–9.20) [27]. In this study, patients with malnutrition were more likely to experience postoperative complications. The multivariate Cox analysis demonstrated that GLIM-defined malnutrition, age, preoperative cerebrovascular disease, and CPB time remain independent risk factors of OS. Preoperative cerebrovascular disease leads to high risk of postoperative stroke and poor long-term outcomes [28]. Prolonged CPB time determines myocardial damage, which could affect mortality directly [29]. The duration of CPB time is correlated with the extent of inflammatory response, while excessive inflammatory response may cause loss of physical capacity and prolonged critical illness, contributing to long-term outcome disadvantages [21]. Thus, four independent risk factors were included to develop the nomogram, which were all independently prognostic for late mortality in previous studies [28,30,31,32].

In comparison with nutritional risk index and screening tools, single clinical indicators such as BMI and albumin could also reflect the nutritional status of patients. It has been reported that BMI and albumin are independent predictors of postoperative complication and mortality after CABG or valve surgery [33,34]. However, Christian et al. suggested that using serum albumin and BMI to recognize malnutrition needs further validation, considering that they are influenced by fluid shifts and systemic inflammation [4]. For the population included in our study, patients with valvular heart disease prone to fluid retention and patients with coronary heart disease are usually overweight. This could conceal low BMI or weight loss that might already exist. As expected, low BMI was not a risk factor for OS in this study. Patients meeting reduced muscle mass accounted for the most significant proportion among the three phenotypic criteria of the GLIM. Compared with albumin, recognizing muscle mass reduction preferably identifies nutritional risk, thus resulting in a better prognostic effect. In consequence, applying the GLIM, which is acknowledged and relatively comprehensive, to patients who underwent cardiac surgery would be helpful to identify malnourished patients and provide specialized nutritional treatment for them.

EuroSCORE II is a recognized tool for predicting in-hospital mortality of patients who underwent cardiac surgery. The prediction efficiency of EuroSCORE II for long-term mortality remains controversial, and its performance fades year by year [35,36]. Although several EuroSCORE II variables were independent risk factors for long-term OS, researchers suggested that they can be used in a different algorithm [36]. Therefore, it is necessary to develop a new risk-stratification tool to predict long-term clinical outcomes. We exploited a nomogram to predict OS more precisely. The nomogram showed the prediction effect on 1-, 2-, and 3-year survival probability, and it was verified in the validation cohort. The nomogram had a bigger AUC than EuroSCORE II, and it showed a better discriminative ability than EuroSCORE II. Additionally, the deviation between the predicted long-term mortality rate of EuroSCORE II did not agree well with the actual mortality rate [35]. The calibration curve of our nomogram revealed good agreement between predicted and actual OS.

This current study has several limitations. The severity of malnutrition was not classified because of the absence of consensus reference cutoff values for muscle mass reductions in the Asian population. Additionally, nutritional status was evaluated only once before surgery. We will focus on the relationship between the changes of postoperative nutritional status and patients’ long-term survival in our further study.

## 5. Conclusions

GLIM criteria can efficiently identify malnutrition and have a prognostic effect on clinical outcomes after cardiac surgery. A GLIM-based nomogram has favorable performance in survival prediction.

## Figures and Tables

**Table 1:** Numbers of patients with malnutrition meeting each GLIM phenotypical criteria.

	Malnutrition, *n* = 162 (18.8%)	Phenotypic Criteria		
		Weight Loss, *n* = 74 (8.6%)	Low BMI, *n* = 29 (3.4%)	Reduced Muscle Mass, *n* = 135 (15.7%)
Training cohort	114(18.9%)	46(7.6%)	18(3.0%)	98(16.3%)
Validation cohort	48 (18.6%)	28 (10.9%)	11 (4.3%)	37 (14.3%)

Abbreviations: BMI: body mass index.

**Table 2:** Preoperative characteristics of study subjects.

	Training Cohort (*n* = 603)			Validation Cohort (*n* = 258)		
	GLIM Criteria			GLIM Criteria		
	Without Malnutrition (*n* = 489)	Malnutrition (*n* = 114)	*p* Value	Without Malnutrition (*n* = 210)	Malnutrition (*n* = 48)	*p* Value
Age, years	63 (11)	65 (11)	0.122	63 (12)	68 (16)	0.020 *
Sex, male	319 (65.2%)	90 (78.9%)	0.005 *	130 (61.9%)	30 (62.5%)	0.939
BMI, kg/m^2^	24.74 (3.66)	21.09 (3.82)	<0.001 *	24.99 (4.24)	22.15 (5.12)	<0.001 *
Tobacco use	179 (36.6%)	41 (36.0%)	0.898	62 (29.5%)	13 (27.1%)	0.737
Alcohol use	67 (13.7%)	15 (13.2%)	0.879	34 (16.2%)	5 (10.4%)	0.314
LVEF, %	60 (8)	60 (9)	0.880	60 (9)	62 (10)	0.636
NYHA class 3 or 4	426 (87.1%)	100 (87.7%)	0.862	184 (87.6%)	44 (91.7%)	0.430
CCI	3 (2)	3 (2)	0.254	3 (2)	3 (2)	0.208
EuroSCORE II	1.64 (1.09)	1.83 (1.50)	0.097	1.65 (0.97)	1.81 (1.27)	0.065
Comorbidities (%)						
Hypertension	338 (69.1%)	67 (58.8%)	0.034 *	136 (64.8%)	30 (62.5%)	0.768
Diabetes	154 (31.5%)	33 (28.9%)	0.597	72 (34.3%)	8 (16.7%)	0.017 *
Chronic heart failure	39 (8.0%)	10 (8.8%)	0.779	18 (8.6%)	6 (12.5%)	0.569
Atrial fibrillation	97 (19.8%)	20 (17.5%)	0.577	38 (18.1%)	8 (16.7%)	0.816
Previous myocardial infarction	21 (4.3%)	6 (5.3%)	0.652	9 (4.3%)	2 (4.2%)	1.000
COPD	15 (3.1%)	5 (4.4%)	0.676	4 (1.9%)	2 (4.2%)	0.684
Recent pneumonia	25 (5.1%)	4 (3.5%)	0.471	9 (4.3%)	3 (6.3%)	0.839
Cerebrovascular disease	61 (12.5%)	16 (14.0%)	0.653	35 (16.7%)	9 (18.8%)	0.729
Laboratory data						
C-reactive protein, mg/L	3.17 (1.44)	3.23 (5.26)	0.020 *	3.17 (1.83)	3.3 (14.66)	0.020 *
White blood cells, ×10^9^/L	6.36 (2.48)	6.43 (2.66)	0.927	6.31 (2.66)	5.67 (3.03)	0.389
Red blood cells, ×10^12^/L	4.37 ± 0.54	4.17 ± 0.58	<0.001 *	4.39 ± 0.56	4.17 ± 0.47	0.013 *
Hemoglobin, g/L	131 (23)	127 (24)	0.023 *	132 (23)	125.5 (16.75)	0.037 *
Platelets, ×10^9^/L	203 (80.5)	199.5 (82.5)	0.939	204.5 (82.75)	202.0 (102.5)	0.626
Neutrophil percentage, %	61.77 ± 9.74	64.41 ± 10.68	0.011 *	62.02 ± 9.62	64.64 ± 11.75	0.155
Lymphocytes, ×10^9^/L	1.73 (0.77)	1.58 (0.69)	0.004 *	1.71 (0.80)	1.49 (0.73)	0.003 *
NLR	2.23 (1.46)	2.44 (2.30)	0.021 *	2.26 (1.46)	2.76 (3.34)	0.064
PLR	113.77 (58.62)	131.12 (81.16)	0.010 *	118.47 (62.81)	143.38 (77.83)	0.025 *
Total protein, g/L	68.76 (7.35)	68.14 (7.03)	0.879	69.00 (7.05)	68.14 (10.13)	0.360
Albumin, g/L	41.1 (5.0)	40.25 (5.0)	0.047 *	41.0 (5.0)	41.0 (6.75)	0.058
BUN, μmol/L	5.9 (2.5)	5.9 (3.0)	0.351	6.1 (2.6)	5.6(2.5)	0.144
Creatinine, μmol/L	76.0 (25.0)	74.1 (28.2)	0.396	76.0 (26.3)	74.8 (28.2)	0.662

Abbreviations: BMI: body mass index; LVEF: left ventricular ejection; NYHA: New York Heart Association; CCI: Charlson Comorbidity Index; EuroSCORE II: European System for Cardiac Operative Risk Evaluation II; COPD: chronic obstructive pulmonary disease; NLR: neutrophil-to-lymphocyte ratio; PLR: platelet-to-lymphocyte ratio; BUN: blood urea nitrogen. * Statistically significant (*p* < 0.05).

**Table 3:** Intraoperative characteristics of study subjects.

	Training Cohort (*n* = 603)			Validation Cohort (*n* = 258)		
	GLIM Criteria			GLIM Criteria		
	Without Malnutrition (*n* = 489)	Malnutrition (*n* = 114)	*p* Value	Without Malnutrition (*n* = 210)	Malnutrition (*n* = 48)	*p* Value
Surgical Type			0.760			0.887
Isolated CABG	277 (56.6%)	65 (57.0%)		110 (52.4%)	27 (56.3%)	
Isolated valve surgery	177 (36.2%)	43 (37.7%)		91 (43.3%)	19 (39.6%)	
CABG + valve surgery	35 (7.2%)	6 (5.3%)		9 (4.3%)	2 (4.2%)	
Operative time, min	215 (61)	211 (57)	0.335	213 (56)	214 (60)	0.790
CPB time, min	81 (54)	80.5 (52)	0.857	82 (41)	76 (52)	0.853
Aortic cross-clamp time, min	59 (31)	61 (25)	0.917	54.5 (28)	60 (35)	0.476
Type of involved valves			0.710			0.712
aortic valve	56 (26.4%)	15 (30.6%)		25 (25.0%)	3 (14.3%)	
mitral valve	55 (25.9%)	14 (28.6%)		21 (21.0%)	3 (14.3%)	
tricuspid	12 (5.7%)	5 (10.2%)		10 (10.0%)	4 (19.0%)	
aortic valve + mitral valve	15 (7.1%)	3 (6.1%)		9 (9.0%)	2 (9.5%)	
aortic valve + tricuspid	5 (2.4%)	1 (2.0%)		0 (0.0%)	0 (0.0%)	
mitral valve + tricuspid	54 (25.5%)	10 (20.4%)		29 (29.0%)	7 (33.3%)	
aortic valve + mitral valve + tricuspid	15 (7.1%)	1 (2.0%)		6 (6.0%)	2 (9.5%)	
CABG details						
CABG type: on pump	202 (64.7%)	52 (73.2%)	0.172	87 (73.1%)	21 (72.4%)	0.940
Use of LIMA	160 (51.3%)	24 (33.8%)	0.008 *	63 (52.9%)	9 (31.0%)	0.034 *
Number of bypassed vessels			0.377			0.320
1	33 (10.6%)	4 (5.6%)		7 (5.9%)	0 (0.0%)	
2	39 (12.5%)	7 (9.9%)		8 (6.7%)	4 (13.8%)	
3	87 (27.9%)	18 (25.4%)		43 (36.1%)	12 (41.4%)	
4 or more	153 (49.0%)	42 (59.2%)		61 (51.3%)	13 (44.8%)	

Abbreviations: CABG: coronary artery bypass grafting; CPB: cardiopulmonary bypass; LIMA: left internal mammary artery. * Statistically significant (*p* < 0.05).

**Table 4:** Details for postoperative outcomes in the training cohort.

	All Patients (*n* = 603)	GLIM Criteria		*p* Value
		Without Malnutrition (*n* = 489)	Malnutrition (*n* = 114)	
Postoperative hospital stay, day	10 (5)	10 (4)	10 (7)	0.126
Prolonged intensive care stay (>5 d)	92 (15.3%)	67 (13.7%)	25 (21.9%)	0.028 *
Indwelling drainage tube time, day	3 (1)	3 (1)	3 (1)	0.503
Cost, CNY	130,926 (44,393)	130,661 (42,484)	131,751 (56,718)	0.084
30 days readmission	35 (5.8%)	26 (5.3%)	9 (7.9%)	0.289
Total Complications	268 (44.4%)	207 (42.3%)	61 (53.5%)	0.031 *
Pneumonia	18 (3.0%)	13 (2.7%)	5 (4.4%)	
Delirium	16 (2.7%)	12 (2.5%)	4 (3.5%)	
Poor wound healing (no debridement)	15 (2.5%)	13 (2.7%)	2 (1.8%)	
Poor wound healing need debridement	15 (2.5%)	11 (2.2%)	4 (3.5%)	
Pleural effusion	93 (15.4%)	74 (15.1%)	19 (16.7%)	
Reoperation	6 (1.0%)	5 (1.0%)	1 (0.9%)	
Stroke	4 (0.7%)	3 (0.6%)	1 (0.9%)	
Low cardiac output syndrome	22 (3.6%)	16 (3.3%)	6 (5.3%)	
Respiratory failure	43 (7.1%)	35 (7.2%)	8 (7.0%)	
MODS	6 (1.0%)	5 (1.0%)	1 (0.9%)	
In-hospital mortality	30 (5.0%)	20 (4.1%)	10 (8.8%)	

Abbreviations: MODS: multiple organ dysfunction syndrome. * Statistically significant (*p* < 0.05).

**Table 5:** Risk factors for postoperative complications in the training cohort.

Factors	Univariate Analysis			Multivariate Analysis		
	OR	95% CI	*p* Value	OR	95% CI	*p* Value
GLIM-defined malnutrition	1.568	1.041–2.361	0.031 *	1.661	1.063–2.594	0.026 *
Age	1.043	1.024–1.063	<0.001 *	1.044	1.024–1.065	<0.001 *
Sex (male)	0.741	0.526–1.044	0.087	0.587	0.402–0.858	0.006 *
BMI < 18.5 kg/m^2^	2.031	0.657–6.281	0.219			
Tobacco use	0.978	0.700–1.365	0.895			
Alcohol use	1.450	0.909–2.313	0.118			
LVEF ≤ 50%	2.332	1.506–3.612	<0.001 *	2.197	1.359–3.552	0.001 *
NYHA class 3 or 4	1.378	0.843–2.254	0.201			
CCI ≥ 2	2.901	1.788–4.707	<0.001 *			
EuroSCORE II ≥ 4%	5.043	2.453–10.367	<0.001 *	2.642	1.231–5.670	0.013 *
Hypertension	1.541	1.088–2.181	0.015 *			
Diabetes	1.450	1.025–2.051	0.036 *			
Chronic heart failure	2.536	1.376–4.677	0.003 *			
Atrial fibrillation	1.187	0.792–1.778	0.407			
Previous myocardial infarction	2.201	0.991–4.890	0.053			
COPD	1.024	0.418–2.507	0.959			
Recent pneumonia	1.359	0.644–2.869	0.420			
Cerebrovascular disease	1.798	1.109–2.915	0.017 *	1.711	1.027–2.851	0.039 *
Hypoproteinemia	1.651	0.805–3.384	0.171			
Surgical Type			0.005 *			
Isolated CABG	1.000	Reference				
Isolated valve surgery	0.647	0.458–0.916	0.014 *			
CABG + valve surgery	1.736	0.895–3.367	0.103			
Operative time, min	1.008	1.005–1.012	<0.001 *	1.008	1.005–1.012	<0.001 *
CPB time, min	1.003	1.000–1.006	0.080			
C-reactive protein > 10 mg/L	1.582	0.988–2.532	0.056			
Hemoglobin, g/L	0.986	0.977–0.995	0.003 *			
NLR ≥ 3.5	1.281	0.870–1.886	0.210			
PLR ≥ 133	1.207	0.864–1.687	0.270			

Abbreviations: BMI: body mass index; LVEF: left ventricular ejection; NYHA: New York Heart Association; CCI: Charlson Comorbidity Index; EuroSCORE II: European System for Cardiac Operative Risk Evaluation II; COPD: chronic obstructive pulmonary disease; CABG: coronary artery bypass grafting; CPB: cardiopulmonary bypass; NLR: neutrophil-to-lymphocyte ratio; PLR: platelet-to-lymphocyte ratio. * Statistically significant (*p* < 0.05).

**Table 6:** Risk factors for overall survival in the training cohort.

Factors	Univariate Analysis			Multivariate Analysis		
	HR	95% CI	*p* Value	HR	95% CI	*p* Value
GLIM-defined malnutrition	2.602	1.687–4.014	<0.001 *	2.339	1.504–3.637	<0.001 *
Age	1.077	1.049–1.105	<0.001 *	1.073	1.046–1.101	<0.001 *
Sex (male)	1.216	0.770–1.921	0.401			
BMI < 18.5 kg/m^2^	1.037	0.255–4.216	0.960			
Tobacco use	0.987	0.642–1.517	0.951			
Alcohol use	1.273	0.730–2.220	0.395			
LVEF ≤ 50%	1.378	0.820–2.314	0.226			
NYHA class 3 or 4	1.444	0.723–2.885	0.298			
CCI ≥ 2	1.709	0.885–3.299	0.110			
EuroSCORE II ≥ 4%	2.679	1.513–4.745	0.001 *			
Hypertension	0.853	0.553–1.315	0.470			
Diabetes	1.405	0.916–2.155	0.119			
Chronic heart failure	1.795	0.928–3.473	0.082			
Atrial fibrillation	1.464	0.911–2.354	0.116			
Previous myocardial infarction	1.064	0.390–2.903	0.903			
COPD	0.296	0.041–2.126	0.226			
Recent pneumonia	0.452	0.111–1.836	0.267			
Cerebrovascular disease	1.906	1.148–3.166	0.013 *	1.980	1.188–3.298	0.009 *
Hypoproteinemia	1.493	0.651–3.420	0.344			
Surgical Type			0.382			
Isolated CABG	1.000	Reference				
Isolated valve surgery	1.357	0.881–2.090	0.166			
CABG + valve surgery	1.112	0.473–2.612	0.808			
Operative time, min	1.006	1.003–1.009	<0.001 *			
CPB time, min	1.013	1.009–1.017	<0.001 *	1.012	1.009–1.015	<0.001 *
C-reactive protein > 10 mg/L	1.172	0.650–2.112	0.598			
Hemoglobin, g/L	0.979	0.967–0.991	<0.001 *			
NLR ≥ 3.5	1.154	0.707–1.883	0.567			
PLR ≥ 133	0.889	0.570–1.384	0.602			

Abbreviations: BMI: body mass index; LVEF: left ventricular ejection; NYHA: New York Heart Association; CCI: Charlson Comorbidity Index; EuroSCORE II: European System for Cardiac Operative Risk Evaluation II; COPD: chronic obstructive pulmonary disease; CABG: coronary artery bypass grafting; CPB: cardiopulmonary bypass; NLR: neutrophil-to-lymphocyte ratio; PLR: platelet-to-lymphocyte ratio. * Statistically significant (*p* < 0.05).

## Data Availability

The data presented in this study are available on request from the corresponding author. The data are not publicly available due to patients’ privacy.

## Supplementary Materials

The following supporting information can be downloaded at: https://www.mdpi.com/article/10.3390/nu14122409/s1, Figure S1: A flow chart of patient selection.
