# Supplementary figures and images for: Development and Validation of Global Leadership Initiative on Malnutrition for Prognostic Prediction in Patients Who Underwent Cardiac Surgery
Source: Nutrients. 2022 Jun 9;14(12):2409. doi: 10.3390/nu14122409 (PMC9230873; doi:10.3390/nu14122409)

**Figure S1.** A flow chart of patient selection.

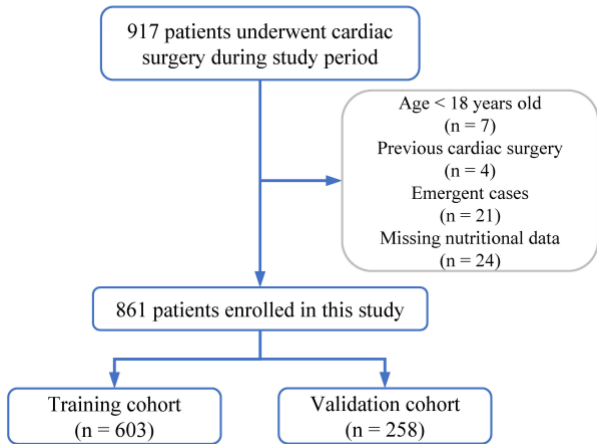

Supplement: Supplementary file 1 [file nutrients-14-02409-s001.zip › nutrients-1714055-supplementary.pdf]
